# Milk yield deviations as indicators of suspected bovine coronavirus outbreaks in Norwegian dairy herds: Evidence from dynamic linear models

**DOI:** 10.3168/jdsc.2026-1059

**Published:** 2026-07-16

**Authors:** Lars Erik Gangsei, Luis Pedro Carmo, Petter Hopp, Gema Vidal, Victor Henrique Silva de Oliveira

**Affiliations:** 1 Norwegian Veterinary Institute, NO-1431 Ås, Norway; 2 Faculty of Chemistry, Biotechnology and Food Science, Norwegian University of Life Sciences (NMBU), NO-1432 Ås, Norway; 3 Veterinary Public Health Institute, Vetsuisse Faculty, University of Bern, 3097 Liebefeld, Switzerland; 4 Swedish Veterinary Agency, Department of Epidemiology, Surveillance and Risk Assessment, 751 89 Uppsala, Sweden

## Abstract

Suspected outbreaks of bovine coronavirus were reported in 35 dairy herds in Møre og Romsdal County, Norway, during autumn 2023 and spring 2024. This study evaluated if these outbreaks could be detected through deviations in bulk tank milk yield. Milk yield was derived from the tank volumes delivered to the dairy company, adjusted for herd size, and modeled using dynamic linear models. During registered suspected outbreaks, milk yield was approximately 2 L per cow per day lower than predicted, and this deviation is assumed to be attributable to bovine coronavirus infection. A simple heuristic threshold of a 2-L reduction in milk yield per cow per day compared with predictions from the dynamic linear models was applied to detect bovine coronavirus outbreaks. Results indicate that such a threshold detects outbreaks but also produces a considerable number of false alarms. Thus, monitoring milk yield at the tank level can reveal production losses associated with suspected bovine coronavirus outbreaks. However, for early warnings before these outbreak periods, analyses that include spatiotemporal patterns across multiple herds will likely be required, as surveillance based on individual-herd data alone resulted in sensitivity and specificity that were lower than desirable.

## Graphical Abstract

**Figure:**
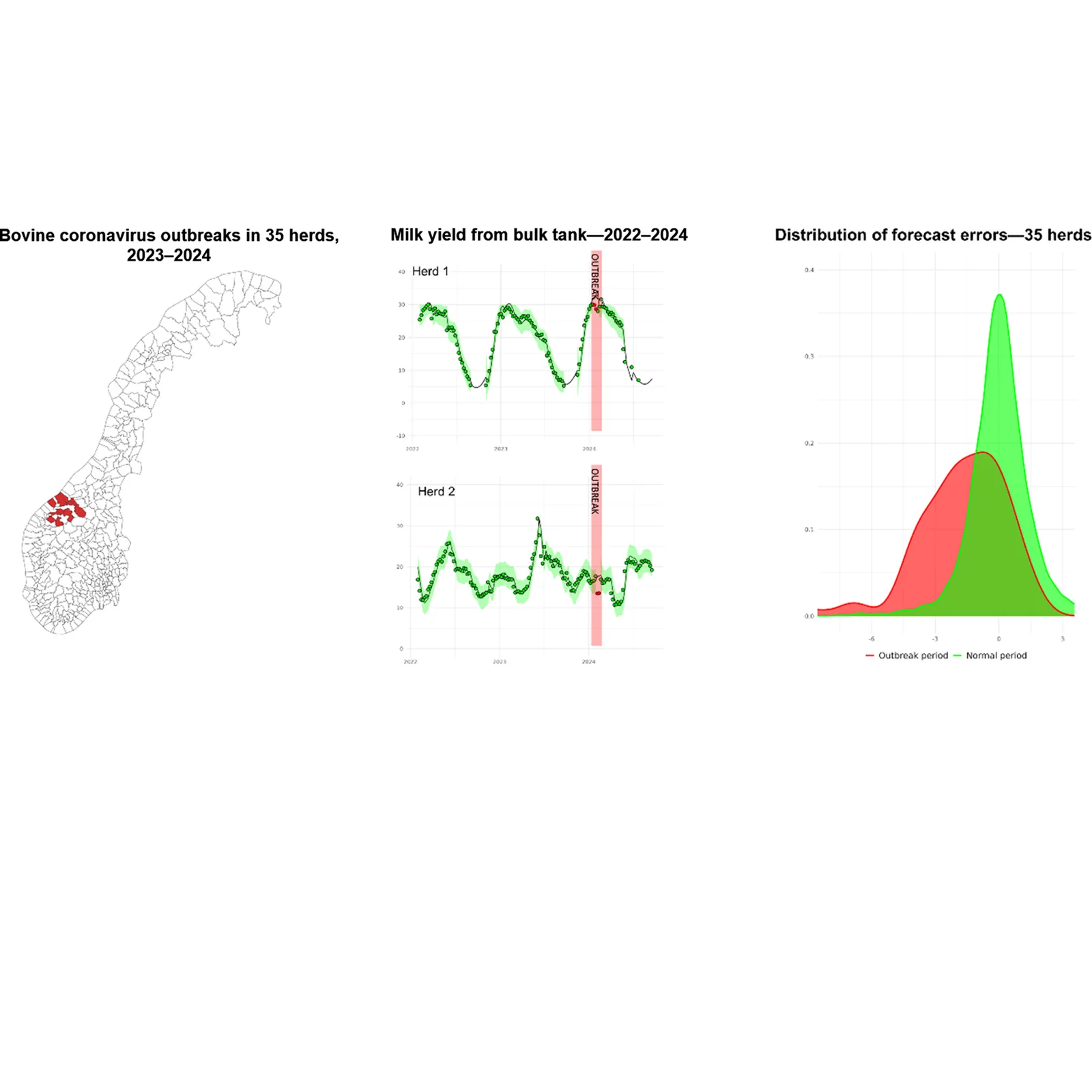

Bovine coronavirus (**BCoV**) is an enveloped, RNA virus of the genus *Betacoronavirus* (ICTV, 2026). It has a worldwide distribution and causes enteric disease in cattle, including neonatal calf diarrhea and winter dysentery in adults (Durham et al., 1989; Oma et al., 2016). Bovine coronavirus is also a recognized component of bovine respiratory disease, and infections are often associated with mixed viral and bacterial pathogens (Oliveira et al., 2020). Transmission occurs mainly through the fecal–oral route and aerosols, with shedding in feces and respiratory secretions, and it can also spread via contaminated surfaces and equipment (Ohlson et al., 2010; Oma et al., 2016). Outbreaks lead to reduced feed intake, decreased milk yield, and impaired growth performance, resulting in economic losses in both beef and dairy production (Toftaker et al., 2017; Vlasova and Saif, 2021).

Effective control strategies for BCoV infection include a combination of strict biosecurity practices, improved hygiene in calving and housing areas, vaccination of pregnant cows to enhance passive immunity in calves, and where relevant, vaccination of calves, along with adequate colostrum intake to ensure passive transfer of immunity in newborns (Vlasova and Saif, 2021). Continuous monitoring of herd health, combined with early isolation of symptomatic animals, reduces viral spread within herds (Vlasova and Saif, 2021). However, despite these established measures, BCoV outbreaks continue to occur, which could be partly explained by the nonspecific and often mild early clinical signs, which can go unnoticed (Durham et al., 1989; Oma et al., 2016), leading to delayed detection and intervention.

In Norway, national dairy production data provide a valuable basis for monitoring herd-level health trends. Bovine coronavirus is considered endemic in Norwegian dairy herds, and infection has been associated with reduced milk yield (Toftaker et al., 2016, 2017). We therefore hypothesized that deviations in bulk tank milk volumes may signal early BCoV infection and might be detected through routine surveillance of milk production data. If confirmed, this approach could support the development of early warning systems for potential disease events and, ultimately, enable timely interventions. The primary aim of the present study was to investigate whether deviations in herd-level milk yield can serve as indicators of suspected BCoV outbreaks in dairy herds, and thereby constitute a basis for outbreak surveillance and possible early warnings to herds at risk. Although quantifying production losses associated with the suspected BCoV outbreaks was not our primary aim, such estimates emerge as a secondary outcome of the study.

The data used in the present study come from 2 different sources: the Norwegian Dairy Herd Recording System (**NDHRS**) and TINE SA, a farmer-owned dairy cooperative. The NDHRS is a national program for recording milk yield and composition, health and fertility traits, and herd management data, used for genetic evaluation, herd benchmarking, and research (Espetvedt et al., 2013). Bulk tank milk volumes measured at dairy herds and reported to NDHRS cover more than 95% of Norwegian dairy herds. TINE SA manages the operational aspects of the NDHRS program, whereas the joint venture company MIMIRO, partly owned by TINE SA, acts as the data controller and database owner.

In addition to the NDHRS data, TINE SA also provided an overview of 35 dairy herds in Møre og Romsdal County, where suspected BCoV outbreaks were reported between November 20, 2023, and March 18, 2024. A suspected outbreak in this dataset was defined as an event reported by veterinarians to TINE SA based on observed signs consistent with BCoV infection. The 35 outbreaks were selected by TINE SA's emergency preparedness and disease surveillance specialists, as they were considered representative examples of BCoV outbreaks in time and space. Although laboratory confirmation was not available for these outbreaks, reporting of suspected BCoV infections is encouraged within the Norwegian cattle industry, including financial incentives for maintaining and documenting BCoV-negative (“green”) herd status (Animalia AS, 2026). To maintain green herd status, herds must be tested regularly (maximum 410 d between tests). The financial incentives likely increase the reliability of clinically reported outbreak data, even though diagnostic testing is only sporadically used to confirm herd virus status.

The dataset included the registration dates for each reported outbreak. The 35 suspected BCoV outbreaks were regarded as observations independent of the NDHRS program. The mean number of bulk tank deliveries per herd and week was 2.59. In 98.9% of the weeks, the number of deliveries was between 2 and 4. To get a regular and consistent time unit for analysis, the volume observations were aggregated into weekly totals for each herd. Data were available from Monday, January 7, 2019, to the week starting Monday, September 16, 2024, totaling 298 wk.

For each week and herd, we then calculated the mean milk yield (liters per cow per day) by dividing the weekly bulk tank milk volume by the number of cows registered in the herd and the number of days in the week. This approach assumes an average yield per cow irrespective of lactation status. The number of cows per week was obtained from NDHRS, and all cows aged ≥2 years (≥731 d) were included. In Norway, the mean age at first calving is approximately 25.7 mo based on national herd data (Geno SA, 2020). If data on the number of lactating cows had been readily available, this measure would have been preferable to reduce noise in the milk yield estimates. Thus, the milk yield definition applied in this study has near complete coverage in Norwegian herds and is therefore highly suitable for surveillance purposes. We assume that this approach is generalizable beyond Norway.

To classify weeks with suspected outbreaks, we applied a heuristic rule. A herd was considered to have a suspected outbreak in a given week if the week's start day (Monday) fell within 9 d before the suspected outbreak's registration date in the TINE SA data or up to 4 d after it. This window is chosen to ensure that exactly 2 consecutive weeks were classified as suspected outbreak weeks for each herd, which is roughly in line with a typical period of substantial milk yield reduction during an outbreak (Toftaker et al., 2017).

The data were analyzed by a dynamic linear model (**DLM**). A DLM consists of 2 parts: a system equation and an observation equation. The system equation describes the development of the true underlying system, whereas the observation equation accounts for random errors that occurs outside what is controlled for by the system (West and Harrison, 1997). In this study the mean milk yield per cow per day was used as the response (observed) variable, with a temporal resolution of one observation per herd per week. Formally, let *Y_it_* be the observed daily milk yield per cow for herd *i* (*i* = 1, . . ., 35) in week *t* (*t* = 1, . . ., 298). The milk yield is assumed to be normally distributed according to the observation equation given by

$$Y_{it}∼N\left(\theta_{it}^{1}+\theta_{it}^{2}+\theta_{it}^{3},\sigma_{i}^{2}\right),$$

where $\sigma_{i}^{2}$ is the herd-specific observation variance. The expected milk yields for the herds and weeks in question are given by an overall mean, $\theta_{it}^{1},$ and 2 seasonal components, $\theta_{it}^{2}$ and $\theta_{it}^{3},$ reflecting a harmonic seasonal pattern.

The expected values evolve over time as defined by the system equation, where the expected values are regarded as a multivariate normally distributed state vector. The system equation contains a herd-specific overall expected yield and herd-specific yearly seasonal component, modeled as a harmonic seasonal pattern (West and Harrison, 1997). Formally the system equation is given as

$$\text{\textbackslash{}[}\left[\begin{array}{c} \theta_{it}^{1}\\ \theta_{it}^{2}\\ \theta_{it}^{3} \end{array}\right]∼N_{3}\left(\left[\begin{array}{ccc} 1 & 0 & 0\\ 0 & \cos\left(\omega \right) & \sin\left(\omega \right)\\ 0 & -\sin\left(\omega \right) & \cos\left(\omega \right) \end{array}\right]\left[\begin{array}{c} \theta_{it-1}^{1}\\ \theta_{it-1}^{1}\\ \theta_{it-1}^{1} \end{array}\right],\left[\begin{array}{ccc} \sigma_{\theta 1}^{2} & 0 & 0\\ 0 & \sigma_{\theta 2}^{2} & 0\\ 0 & 0 & \sigma_{\theta 2}^{2} \end{array}\right]\;\right).\text{\textbackslash{}]}$$

There were 3 unknown variance components in the DLM that had to be estimated. Two of these were associated with the system equation, where variance components were assumed to be the same over all herds. Specifically, the variance of the overall mean yield at the herd level, $\sigma_{\theta 1}^{2},$ and the variance of the harmonic seasonal component, $\sigma_{\theta 2}^{2},$ had to be estimated. The third variance component was the mean error variance, σ^2^. We assumed that the herd-specific observation variances, $\sigma_{i}^{2},$ were proportional to the variances of the detrended milk yield time-series, leaving the mean error variance, σ^2^, as the sole unknown parameter shared across all herds for the observation equation. Maximum likelihood estimates were used for the variance components by applying the Kalman filter to the DLM (West and Harrison, 1997).

A major attribute of the DLM is its ability to forecast the milk yield (i.e., to predict the milk yield for the following week based on all data observed up to the week in question). The forecast error is defined as the difference between the observed milk yield 1 wk ahead and the corresponding forecast. These forecast errors should be Gaussian-distributed with zero mean and with an error variance determined by a combination of the observation equation and system equation variances. The main hypothesis in the current study is that forecast errors in periods of suspected BCoV outbreaks should diverge from forecast errors in periods without such outbreaks. Specifically, during outbreak periods we expect the forecast errors to be negative; that is, the observed milk yield should be lower than the yield predicted based on observations before the outbreak.

To examine whether this hypothesis was supported, we omitted data from suspected outbreak weeks when fitting the model. We then ran the DLM and calculated forecast errors for both suspected outbreak periods (i.e., the 2 consecutive outbreak weeks for each herd) and non-outbreak periods. We compared the distributions of forecast errors in outbreak periods and non-outbreak periods. The degree to which these distributions diverge is crucial for assessing the potential of using deviations in milk yield to predict suspected BCoV outbreaks. Because the analysis compares outbreak and non-outbreak periods within the same herds, herds without outbreaks were not required for evaluating model performance.

The mean study herd size was 33.5 cows with 10th, 50th (median), and 90th percentiles of 15, 30, and 55 cows, respectively. These herds had a mean milk yield per cow per day of 22.1 L, with a standard deviation of 5.8 L and 10th, 50th, and 90th percentiles of 14.9, 22.1, and 28.7 L, respectively, after truncation of daily yields below 5 L and above 50 L. Herds with a mean milk yield per cow exceeding 50 L were either those with a very limited number of cows for the week in question or herds with assumed inconsistencies in the registered bulk tank milk volume. Herds with a mean milk yield per cow below 5 L were assumed to represent herds where most cows were dried off and were therefore not representative.

Figure 1 presents the empirical distributions of the forecast errors in periods with suspected outbreaks and non-outbreak periods. The figure clearly shows that the forecast errors in periods with no registered outbreaks have a distribution that is close to Gaussian with a zero mean. The empirical mean is 0.13, and the empirical standard deviation is 2.21. During outbreak periods we observed an empirical distribution with a mean of −1.89 L, with 5%, 50%, and 95% percentiles at −5.71, −1.52, and 1.05 L, respectively, and a standard deviation of 2.55. The distribution appears slightly left-skewed. Its mean differs significantly from zero (*P* < 0.001, *t* = −6.181, df = 69). It is worth noting that the forecast error in outbreak periods can be interpreted as the milk yield loss due to suspected BCoV outbreaks. The mean value of −1.89 L per cow per day is in line with the literature (e.g., Toftaker et al., 2017), which reported a mean loss of 51 L over 26 d, corresponding to approximately 2 L per day.

**Figure 1:**
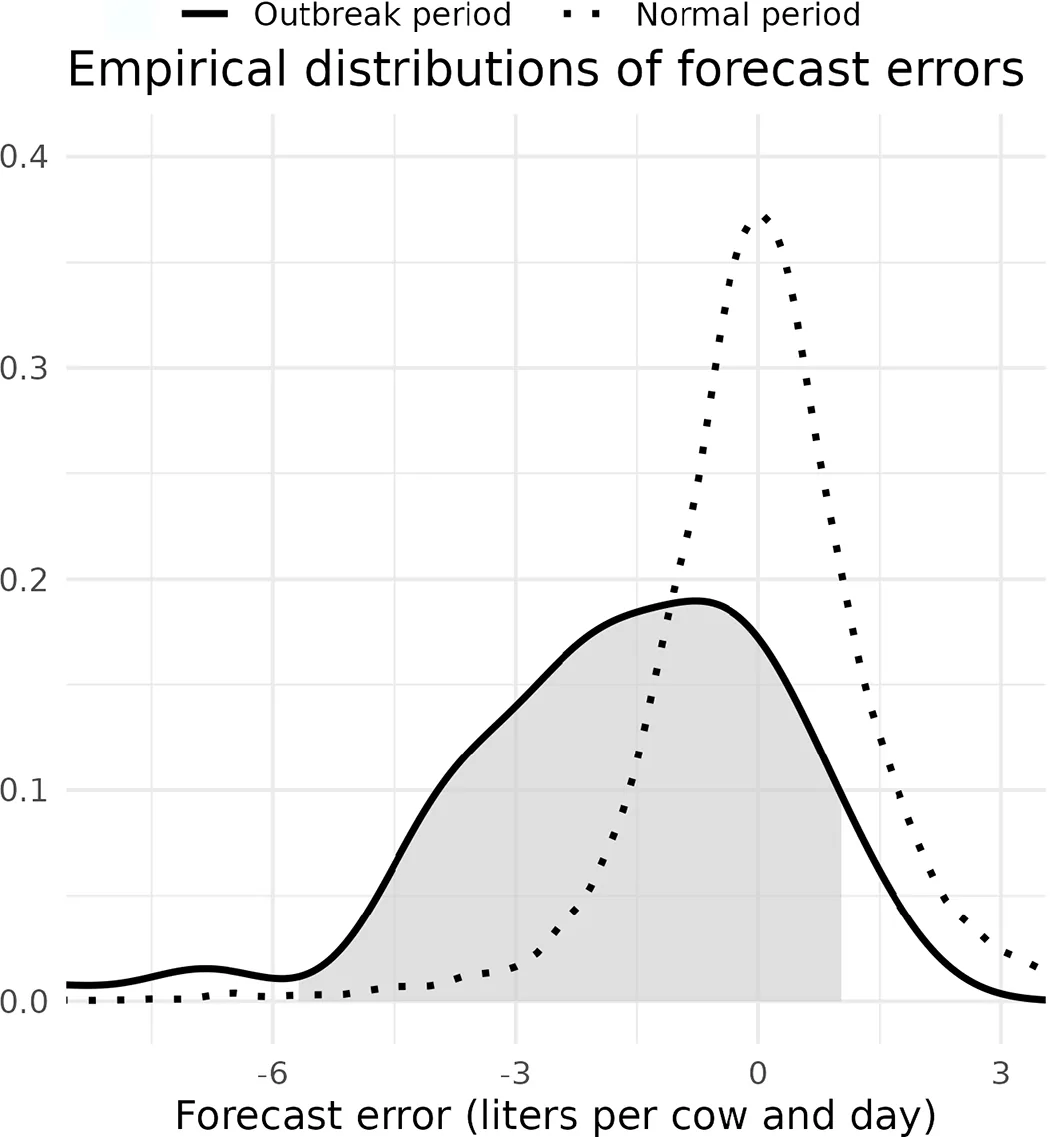
Empirical densities for forecast errors across all herds and over the full study period. The solid black line represents the forecast errors from weeks classified as outbreak periods (n = 70). The shaded area shows the 5% and 95% percentile range of forecast errors during outbreak periods. The dotted line represents the empirical distribution of forecast errors in non-outbreak periods (n = 9,937; i.e., the periods in which no bovine coronavirus outbreak was reported).

Even though the distribution of forecast errors in suspected outbreak periods differs clearly from the distribution of forecast errors in non-outbreak periods (cf. Figure 1), there is considerable overlap between the 2. If we applied a forecast error of less than −2 L as a simple heuristic decision rule for a suspected outbreak, this rule results in a sensitivity of 30/70 (43%; i.e., the proportion of outbreak periods correctly flagged), and a specificity of 9,339/9,937 (94%; i.e., the proportion periods correctly classified without outbreaks). Out of the 35 herds, a subset of 20 (57%) had a forecast error of less than −2 L (i.e., was “flagged” in at least one of the 2 outbreak weeks).

Figure 2 shows the time pattern for 2 different herds. The herd represented in the upper panel shows a pattern where the milk yield in the 2 outbreak weeks is well below the forecasts, indicating that the outbreak could potentially be detected based on the reduced milk yield. In contrast, the herd represented in the lower panel shows no such deviations, as production during the outbreak remains in line with the forecasts.

**Figure 2:**
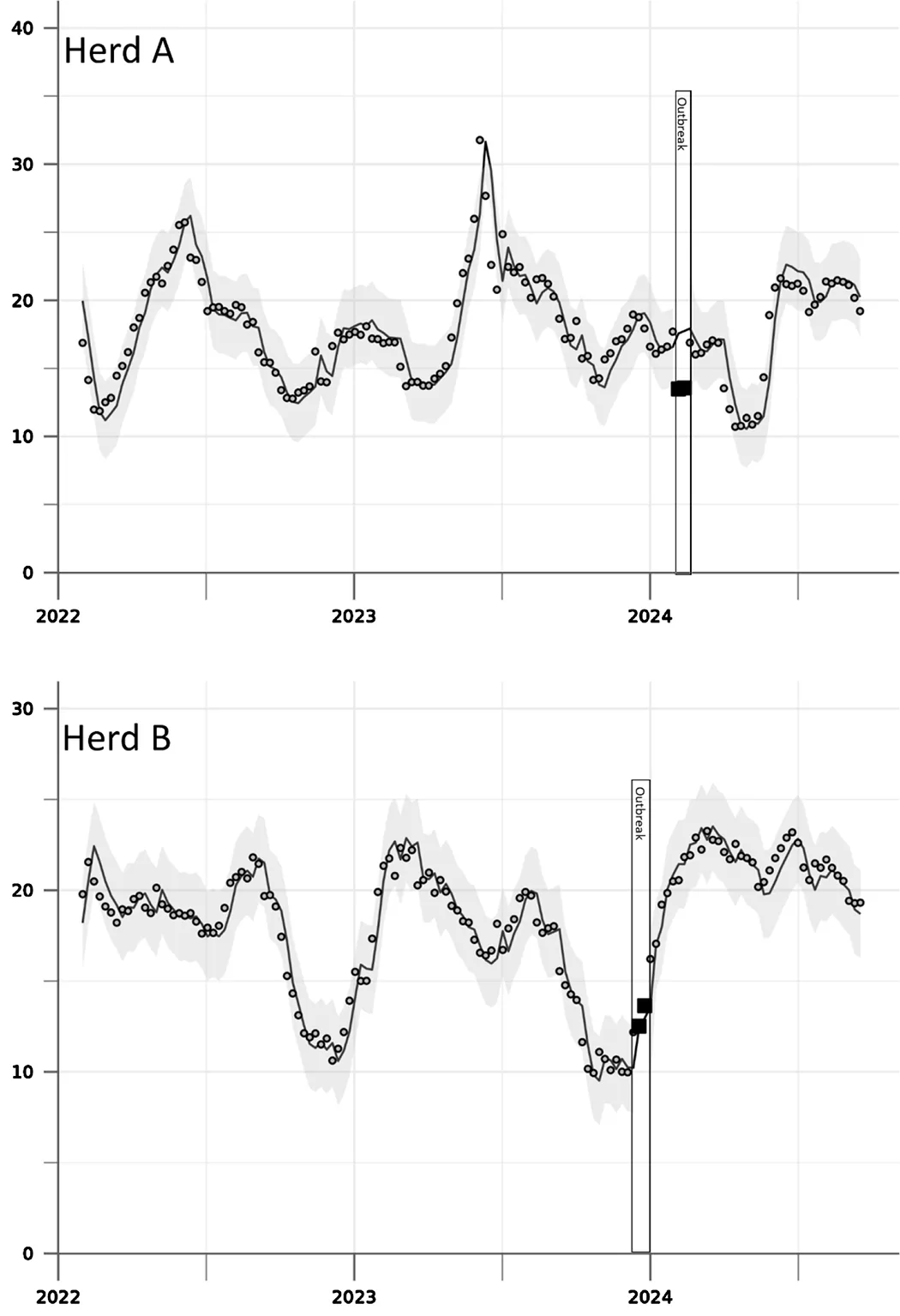
Mean milk yield for 2 herds in the period January 2022 till September 2024. Gray dots indicate weekly mean values for milk yield (liters per cow and day), black lines show the forecast from the dynamic linear model, and the shaded gray area indicates a 95% prediction interval for the forecasts. Mean yield for the 2 outbreak weeks is shown with large black squares, and the time period of the outbreak is indicated with a box.

The results clearly indicate that the deviations in bulk tank milk yield contain information about the suspected BCoV outbreaks. However, the sensitivity obtained using the heuristic rule is relatively low at 43%. The specificity at 94% is also lower than desirable, as it results in a large number of “false alarms.” It should be noted, however, that many of these apparent “false alarms” may in fact represent real events, as it is possible, indeed likely, that these herds experienced unregistered BCoV outbreaks or other endemic illnesses that could have decreased milk yield during the study period. The sensitivity and specificity of the models may also have been influenced by data constraints, including reliance on clinically reported outbreaks rather than laboratory-confirmed BCoV status, as well as unaccounted variability in milk production related to herd dynamics such as the introduction of newly calved cows in early lactation.

Based on the results of the current study, we hypothesize that bulk tank milk yield data could be used as a warning signal, potentially even an early warning, for BCoV outbreaks. The use of BCoV in the present study is mainly heuristic, as we had access to independent information on herds and dates with suspected outbreaks. The method and rationale could also be applied to outbreaks of other transmissible diseases, as well as to noninfectious conditions such as drought or extreme temperatures that lead to production losses and reduced animal health. To strengthen both the sensitivity and specificity of such analyses, models capable of addressing the spatiotemporal nature of disease dynamics will likely be required. Future work could focus on extending the current framework to include explicit spatiotemporal structure, for instance by combining Besag-type spatial random effects (Besag et al., 1991) with temporal processes. This may improve early warning performance by exploiting correlations between nearby herds and temporal persistence in disease dynamics.
